# Supplementary material for: Tunable cell differentiation via reprogrammed mating-type switching
Source: Nat Commun. 2024 Sep 17;15:8163. doi: 10.1038/s41467-024-52282-w (PMC11408693; doi:10.1038/s41467-024-52282-w)
Supplement: Supplementary file 1 — Supplementary Information [file 41467_2024_52282_MOESM1_ESM.pdf]

Supplementary Information

Tunable Cell Differentiation via Reprogrammed Mating-Type Switching

Yu Chyuan Heng[1,2], Shohei Kitano[1,2,3,4], Adelia Vicanatalita Susanto[1,2,3,4], Jee Loon Foo[1,2,3,4*], and Matthew Wook Chang[1,2,3,4*]

[1]Department of Biochemistry, Yong Loo Lin School of Medicine, National University of Singapore, Singapore, Singapore.
[2]NUS Synthetic Biology for Clinical and Technological Innovation (SynCTI), National University of Singapore, Singapore, Singapore.
[3]Synthetic Biology Translational Research Programme, Yong Loo Lin School of Medicine, National University of Singapore, Singapore, Singapore.
[4]National Centre for Engineering Biology (NCEB), Singapore.

[*]Correspondence:

Jee Loon Foo, jeeloon.foo@nus.edu.sg
Matthew Wook Chang, bchcmw@nus.edu.sg

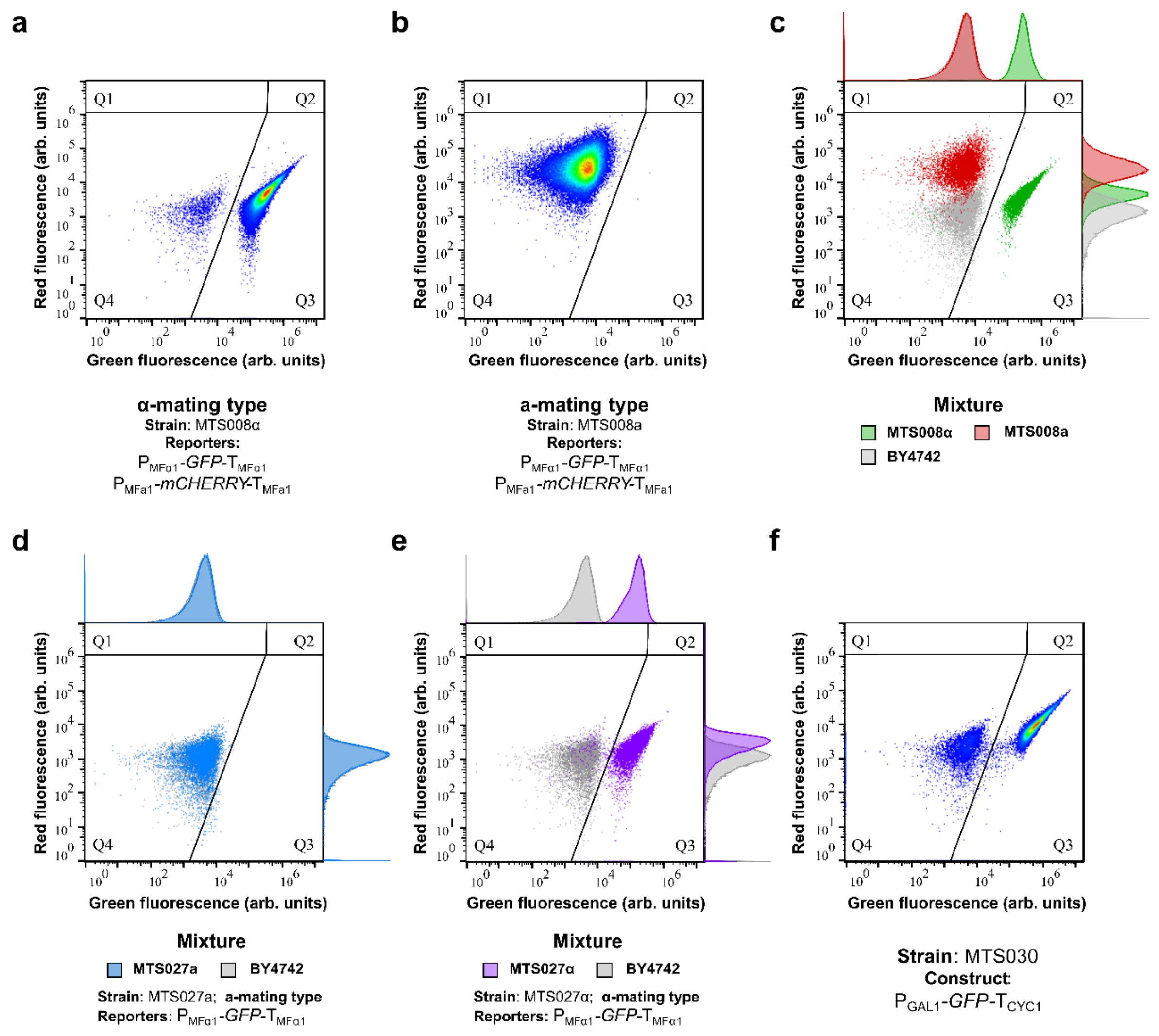

Figure S1 | Development of fluorescent reporter system through the integration of *GFP* and *mCHERRY* genes into the *mfα1*Δ and *MFa1* loci, respectively. (a) Fluorescence scatter plot of strain MTS008α, an α-mating type derivative of the sterile strain MTS007, which was unable to switch mating type due to a recessive *ho* gene and contained both the *GFP* and *mCHERRY* reporter constructs. Two populations were observed: a dominant population comprising 97.37% cells in Q3 and a subpopulation comprising 2.63% cells in Q4 (average values from 144 independent samples). **(b)** Fluorescence scatter plot of strain MTS008a, an a-mating type equivalent of strain MTS008α, showing a single population in Q4. **(c)** Overlap of the fluorescence scatter plots of strains MTS008α (green), MTS008a (red), and BY4742 (grey). The fluorescence distribution of the subpopulation in strain MTS008α was comparable to that of strain BY4742. This suggested that cells within the subpopulation emitted only autofluorescence, without green or red fluorescence from the reporter constructs. **(d)** Fluorescence scatter plot of strain MTS027a (blue), a related strain to MTS008a but lacking the *mCHERRY* reporter construct, showing a single population in Q4 with a fluorescence profile similar to that of strain BY4742. The absence of a population in Q3 indicated that *GFP* expression was exclusive to the α-mating type haploids. **(e)** Fluorescence scatter plot of strain MTS027α (purple), an α-mating type equivalent of MTS027a, showing a similar subpopulation in Q4. This suggested that the presence of the subpopulation was unrelated to the *mCHERRY* reporter construct. **(f)** Fluorescence scatter plot of strain MTS030, a sterile strain expressing

GFP from the strong *GAL1* promoter upon induction with 2% galactose. A similar subpopulation was observed in Q4, indicating its presence was unrelated to moderate *GFP* expression from the *MFα1* promoter in the *GFP* reporter construct. Overall, it was clear that the subpopulation in Q4, which was consistently observed in all α-mating type strains, was part of the α-mating type population comprising of haploids without green fluorescence. Excluding this subpopulation, the α- and a-mating type haploids could be readily distinguished on a fluorescence scatter plot: α-mating type haploids exhibited high green fluorescence and above-control red fluorescence in Q3, and a-mating type haploids exhibited no green fluorescence but high red fluorescence in Q4. The above-control red fluorescence observed in the α-mating type haploids was due to spillover signal from GFP. This occurred because the flow cytometer used a blue laser (488 nm) to excite both GFP and mCherry, and the signal from GFP could be detected by both the green fluorescence (FL1; 520/30 nm) and red fluorescence (FL4; 610/20 nm) channels, albeit sub-optimally by the latter. The percentage of α-mating type haploids was determined by dividing the percentage of cells in Q3 by 97.37%. The percentage of a-mating type haploids was calculated by subtracting the calculated percentage of α-mating type haploids from 100%. All fluorescence plots were generated by flow cytometry after incubating the respective strains in a medium containing 2% glucose for 24 hours, except MTS030, which was grown in a medium containing 2% galactose.

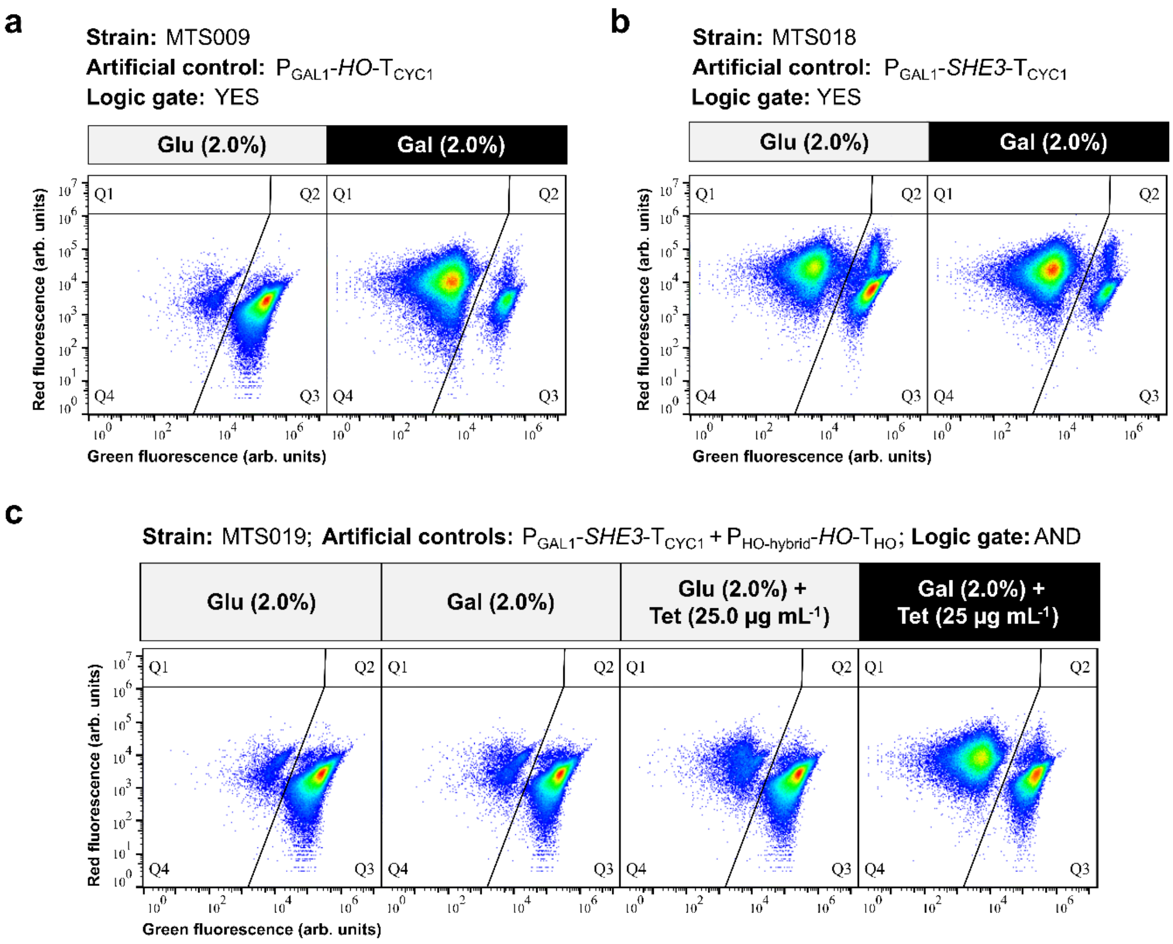

Figure S2 | Development of genetic logic gates for tunable cell differentiation to form synthetic microbial consortia. (a) Fluorescence scatter plot of strain MTS009, a sterile strain integrated with the P_{GAL1}-*HO*-T_{CYC1} module at the recessive *ho* locus. **(b)** Fluorescence scatter plot of strain MTS018, a sterile strain integrated with the P_{GAL1}-*SHE3*-T_{CYC1} module at the *SHE*3 locus and with the recessive *ho* gene replaced with the dominant *HO* gene. **(c)** Fluorescence scatter plot of strain MTS019, a derivative of strain MTS018 with the leaky *HO* promoter replaced with a hybrid promoter, $P_{HO\text{-}hybrid}$, and the P_{TEF1}-*TetR*-T_{CYC1} expression module integrated at the *mfα*2Δ locus. All fluorescence plots were generated using flow cytometry after cultivating the respective strains in a medium containing either glucose (2%), galactose (2%) and/or tetracycline (25 μg mL^{-1}) for 24 hours, followed by dilution and two daily passage in fresh medium containing 2% glucose.

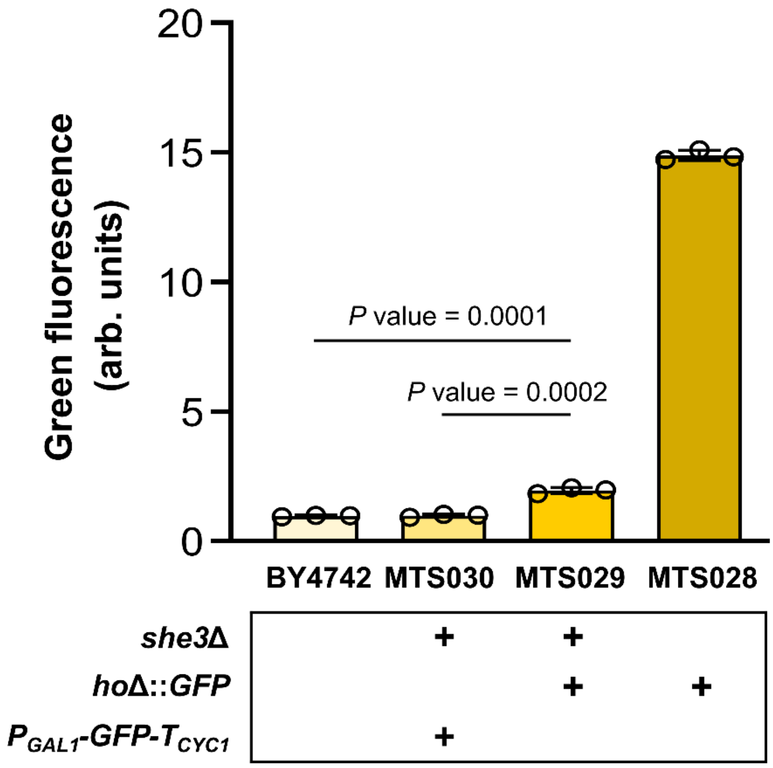

Figure S3 | *GFP* expression from the *HO* promoter. Strain MTS029, with the *SHE3* gene and consequently the *ASH1* mRNA asymmetric transport system disrupted, exhibited leaky *GFP* expression from the *HO* promoter at a level approximately two-fold higher than that of strains BY4742 and MTS030. MTS030 was a strain with repressed *GFP* expression from the *GAL1* promoter, while MTS028 was a control strain with an intact *SHE3* gene. All strains were grown in medium containing 2% glucose for 24 hours before green fluorescence quantification using flow cytometry. Values shown represent the mean ± standard deviation. n=3 biological replicates. Statistical significances were determined using two-tailed Student's t-tests with 95% confidence intervals. Source data are provided as a Source Data file.

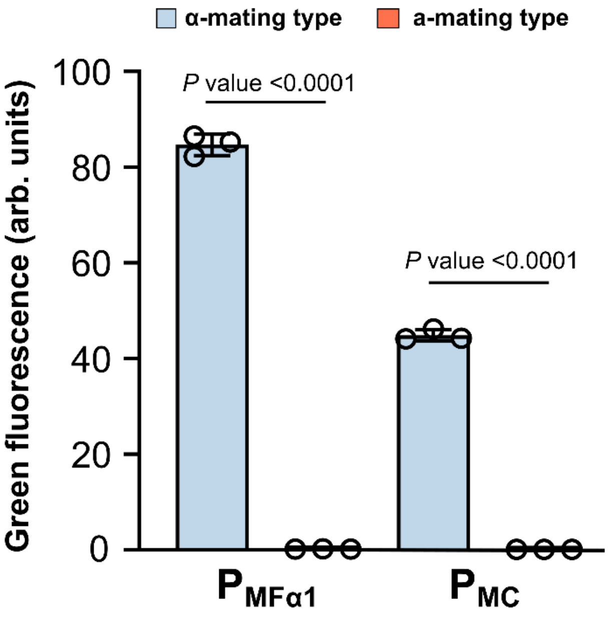

Figure S4 | Characterization of the mating-type-specificity and expression strength of the hybrid promoter, P_{MC}. The P_{MC} promoter was constructed by replacing the TATA box-containing sequence downstream of the $P_{MF\alpha1}$ (-275 to -1 bp) with that from the P_{CYC1} (-252 to -1 bp), while keeping the upstream sequence (-1000 to -276 bp) containing the P'Q elements essential for α-mating-type-specific expression intact. P_{MC} retained α-mating type specificity, but its expression strength was half that of $P_{MF\alpha1}$. Values shown represent the mean ± standard deviation. n=3 biological replicates. Statistical significances were determined using two-tailed Student's t-tests with 95% confidence intervals. Source data are provided as a Source Data file.

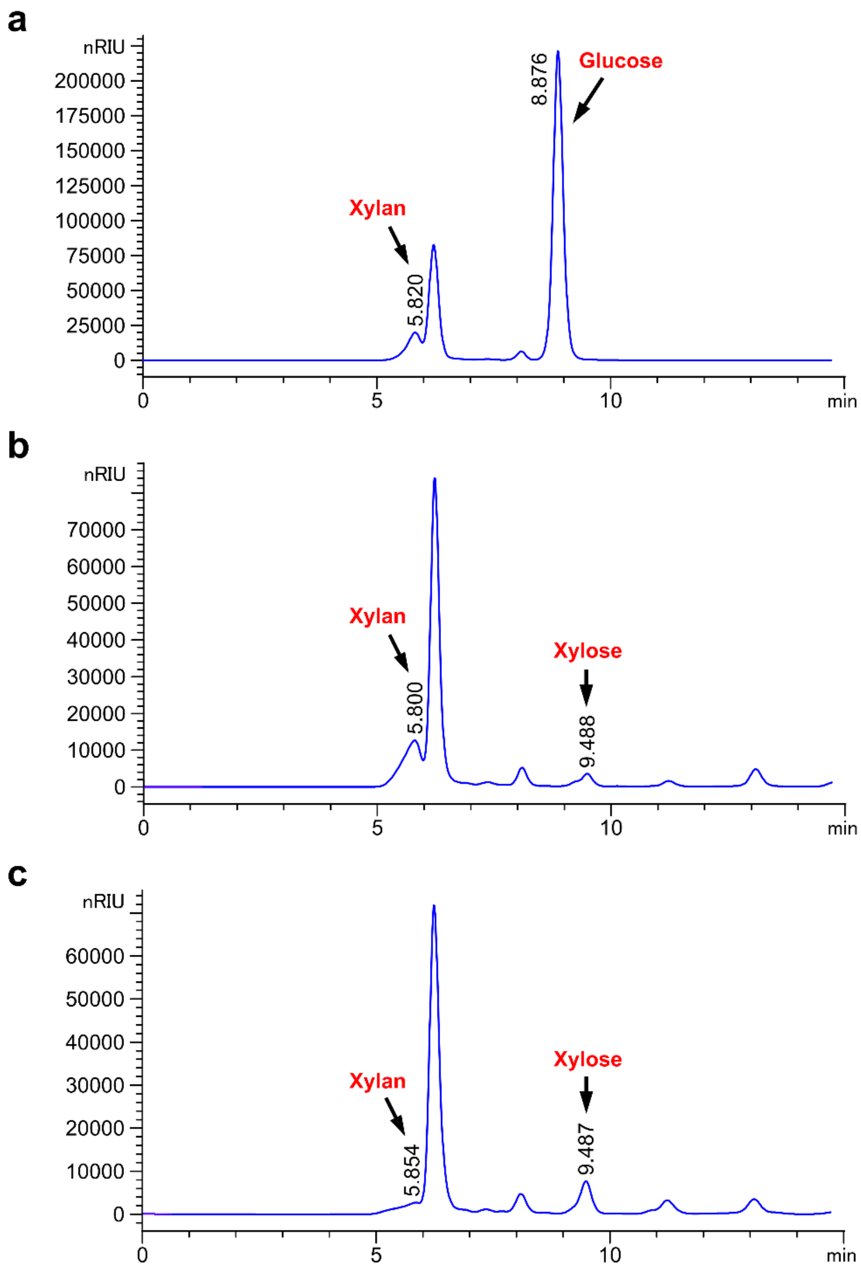

Figure S5 | Representative HPLC chromatograms of **(a)** biotransformation medium containing 1% xylan and supernatant of a microbial consortium incubated in the same biotransformation medium for **(b)** 24 hours and **(c)** 48 hours. The microbial consortium comprised of haploids of opposite mating types, each expressing an enzyme of the xylanolytic pathway. Xylose was produced from xylan through the sequential activity of the XynII and XylA enzymes.

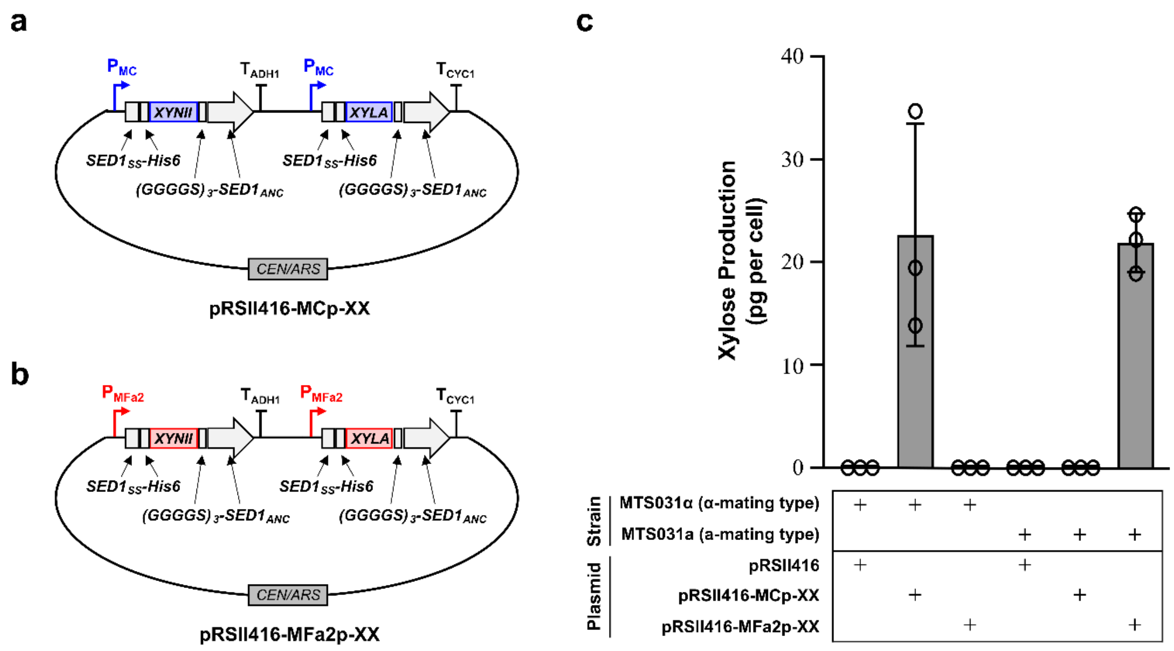

Figure S6 | Single-strain monocultures for xylose bioproduction. (a) pRSII416-MCp-XX and **(b)** pRSII416-MFa2p-XX were plasmid derivatives of pRSII416-XynII-XylA. The former utilized P_{MC} promoter to express both XynII and XylA xylanolytic enzymes, while the latter employed P_{MFa2} promoter. Consequently, enzyme expression was restricted to α-mating type haploids for the former, and a-mating type haploids for the latter. **(c)** Single-strain monocultures were established by transforming strains MTS031α and MTS031a with pRSII416-MCp-XX and pRSII416-MFa2p-XX, respectively. Strains MTS031α and MTS031a were α- and a-mating type derivatives of strain MTS019, which were unable to switch mating type due to a recessive *ho* gene. The transformant strains were incubated in medium containing 1% xylan for 24 hours to examine their efficiency in xylose bioproduction. Four other strains were included as controls, which did not produce xylose from xylan. Values shown in panel (C) represent the mean ± standard deviation. n=3 biological replicates. Source data are provided as a Source Data file.

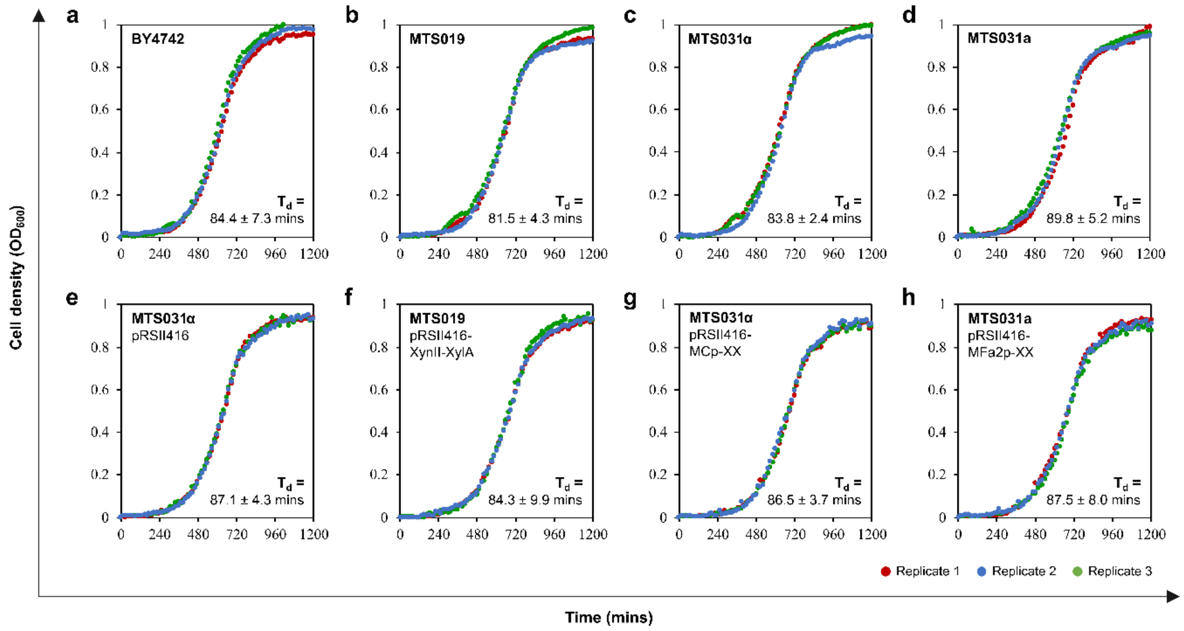

Figure S7 | Characterization of the growth rates of strains MTS019, MTS031α, MTS031a and their transformant derivatives harboring either the pRSII416-XynII-XylA, pRSII416-MCp-XX, or pRSII416-MFa2p-XX plasmid. Strains BY4742 and MTS031α pRSII416 were included as controls. Overnight seed cultures of the respective strains were inoculated at an initial OD_{600} of 0.1 in synthetic defined medium or synthetic minimal medium lacking uracil (for transformant strains) containing 2% glucose. The samples were then loaded at a volume of 100 µL onto a 96-well microplate and subjected to a 20-hour kinetic analysis using a plate reader. The cells were incubated at 30 °C, 807 cpm, with OD_{600} measured at a 15-minute interval. Doubling time (T_d) was calculated by dividing Ln(2) by the exponent of cell density versus time during the exponential phase (135 to 660 minutes). The doubling time value shown in each panel represents the mean ± standard deviation. n=3 biological replicates. Source data are provided as a Source Data file.
