# Supplementary material for: Tunable cell differentiation via reprogrammed mating-type switching
Source: Nat Commun. 2024 Sep 17;15:8163. doi: 10.1038/s41467-024-52282-w (PMC11408693; doi:10.1038/s41467-024-52282-w)
Supplement: Supplementary file 2 — Reporting Summary [file 41467_2024_52282_MOESM2_ESM.pdf]

nature portfolio

Corresponding author(s): Chang, Matthew Wook

Last updated by author(s): Aug 30, 2024

Reporting Summary

Nature Portfolio wishes to improve the reproducibility of the work that we publish. This form provides structure for consistency and transparency in reporting. For further information on Nature Portfolio policies, see our Editorial Policies and the Editorial Policy Checklist.

Statistics

For all statistical analyses, confirm that the following items are present in the figure legend, table legend, main text, or Methods section.

n/a	Confirmed	
☐	☒	The exact sample size (n) for each experimental group/condition, given as a discrete number and unit of measurement
☐	☒	A statement on whether measurements were taken from distinct samples or whether the same sample was measured repeatedly
☐	☒	The statistical test(s) used AND whether they are one- or two-sided<br>*Only common tests should be described solely by name; describe more complex techniques in the Methods section.*
☒	☐	A description of all covariates tested
☒	☐	A description of any assumptions or corrections, such as tests of normality and adjustment for multiple comparisons
☐	☒	A full description of the statistical parameters including central tendency (e.g. means) or other basic estimates (e.g. regression coefficient) AND variation (e.g. standard deviation) or associated estimates of uncertainty (e.g. confidence intervals)
☐	☒	For null hypothesis testing, the test statistic (e.g. F, t, r) with confidence intervals, effect sizes, degrees of freedom and P value noted<br>*Give P values as exact values whenever suitable.*
☒	☐	For Bayesian analysis, information on the choice of priors and Markov chain Monte Carlo settings
☒	☐	For hierarchical and complex designs, identification of the appropriate level for tests and full reporting of outcomes
☒	☐	Estimates of effect sizes (e.g. Cohen's d, Pearson's r), indicating how they were calculated

Our web collection on statistics for biologists contains articles on many of the points above.

Software and code

Policy information about availability of computer code

Data collection	BD Accuri C6, Leica Application Suite X
Data analysis	FlowJo v10.10

For manuscripts utilizing custom algorithms or software that are central to the research but not yet described in published literature, software must be made available to editors and reviewers. We strongly encourage code deposition in a community repository (e.g. GitHub). See the Nature Portfolio guidelines for submitting code & software for further information.

Data

Policy information about availability of data

All manuscripts must include a data availability statement. This statement should provide the following information, where applicable:

- Accession codes, unique identifiers, or web links for publicly available datasets
- A description of any restrictions on data availability
- For clinical datasets or third party data, please ensure that the statement adheres to our policy

All data generated in this study are provided in the Source Data file. Source data are provided with this paper.

Research involving human participants, their data, or biological material

Policy information about studies with human participants or human data. See also policy information about sex, gender (identity/presentation), and sexual orientation and race, ethnicity and racism.

Reporting on sex and gender	NA
Reporting on race, ethnicity, or other socially relevant groupings	NA
Population characteristics	NA
Recruitment	NA
Ethics oversight	NA

Note that full information on the approval of the study protocol must also be provided in the manuscript.

Field-specific reporting

Please select the one below that is the best fit for your research. If you are not sure, read the appropriate sections before making your selection.

☒ Life sciences ☐ Behavioural & social sciences ☐ Ecological, evolutionary & environmental sciences

For a reference copy of the document with all sections, see nature.com/documents/nr-reporting-summary-flat.pdf

Life sciences study design

All studies must disclose on these points even when the disclosure is negative.

Sample size	All experiments were repeated with independent biological replicates at least three times as stated in the figure legends.
Data exclusions	None
Replication	All experiments were repeated with independent biological replicates at least three times as stated in the figure legends.
Randomization	Colonies were randomly picked.
Blinding	N/A

Reporting for specific materials, systems and methods

We require information from authors about some types of materials, experimental systems and methods used in many studies. Here, indicate whether each material, system or method listed is relevant to your study. If you are not sure if a list item applies to your research, read the appropriate section before selecting a response.

Materials & experimental systems

n/a	Involved in the study	
☒	☐	Antibodies
☒	☐	Eukaryotic cell lines
☒	☐	Palaeontology and archaeology
☒	☐	Animals and other organisms
☒	☐	Clinical data
☒	☐	Dual use research of concern
☒	☐	Plants

Methods

n/a	Involved in the study	
☒	☐	ChIP-seq
☐	☒	Flow cytometry
☒	☐	MRI-based neuroimaging

Plants

Seed stocks: NA

Novel plant genotypes: NA

Authentication: NA

Flow Cytometry

Plots

Confirm that:

☒ The axis labels state the marker and fluorochrome used (e.g. CD4-FITC).

☒ The axis scales are clearly visible. Include numbers along axes only for bottom left plot of group (a 'group' is an analysis of identical markers).

☒ All plots are contour plots with outliers or pseudocolor plots.

☒ A numerical value for number of cells or percentage (with statistics) is provided.

Methodology

Sample preparation

To characterize the expression strength of mating-type-specific promoters, overnight seed cultures of the GFP-integrated strains, MTS020-MTS025, MTS026α, MTS026a, MTS027α, and MTS027a, were resuspended to an initial OD600 of 0.2 in synthetic defined medium containing 2% glucose. The starting cultures were loaded at a volume of 100 µL onto a 96-well microplate and cultivated at 30 °C, 999 rpm for 24 hours before flow cytometry measurement. Strains MTS028-MTS030 (and the control BY4742) were similarly prepared and incubated in synthetic defined medium containing 2% glucose for the examination of leaky GFP expression from the HO promoter. Fluorescence scatter plots of strains BY4742, MTS008α, MTS008a, MTS027α, MTS027a, and MTS030 were generated using cells similarly prepared except that the MTS030 strain was incubated in synthetic defined medium containing 2% galactose.

For the characterization of the two YES and the AND logic gates, starting cultures of strains MTS009, MTS018, and MTS019 were similarly prepared but treated with different combinations of glucose (0-2%), galactose (0-8%) and tetracycline (0-100 µg mL-1). Cells were cultivated at a volume of 100 µL on a 96-well microplate, at 30 °C, 999 rpm. The cultures, grown after 24 hours of treatment (1 day of treatment), were subjected to two rounds of daily passage (5 µL of culture diluted) in 100 µL of synthetic defined medium containing 2% glucose (2 days of recovery) before flow cytometry measurement. This allowed sufficient time for the degradation of fluorescent proteins produced by the previous mating type in the absence of inducers. The induction timing was set to 24 hours because, by this duration, the cells would reach saturation (Figure S7). To examine the stability of the population generated by the AND logic gate, cultures of strain MTS019 formed after 1 day of treatment and 2 days of recovery were further passaged for an additional four days in synthetic defined medium containing 2% glucose. For the sequential induction assays, cultures of strain MTS019 were subjected to three rounds of the 1-day of treatment plus 2-day of recovery procedures. For the continuous induction assays, cultures of strain MTS019 were incubated in the same medium for an additional two days, with daily samplings similarly done by first passaging the grown cultures two rounds in synthetic defined medium containing 2% glucose before flow cytometry measurement.

All samples were cultivated in a 96-well microplate using a Multitron Pro microplate shaker (Infors HT). All samples were diluted appropriately with deionized water prior to fluorescence measurement using a BD AccuriTM C6 flow cytometer equipped with a blue laser (488 nm) and a BD CSamplerTM. Typically, 100,000 cells were analysed at a flow rate of 35 µL min-1 (core size 16 µm) to estimate the mean fluorescence or the population composition of a sample. Green fluorescence and red fluorescence were detected by the FL1 (520/30 nm) and FL4 (610/20 nm) channels, respectively. All data acquired were analysed using FlowJo v10.10 software (BD Biosciences).

Instrument: BD Accuri C6

Software: FlowJo v10.10

Cell population abundance: The percentage of the different cell populations are provided in the manuscript.

Gating strategy: The cells are gated based on differential expression of the GFP and mCherry fluorescent proteins.

☒ Tick this box to confirm that a figure exemplifying the gating strategy is provided in the Supplementary Information.
