# Supplementary material for: Tunable cell differentiation via reprogrammed mating-type switching
Source: Nat Commun. 2024 Sep 17;15:8163. doi: 10.1038/s41467-024-52282-w (PMC11408693; doi:10.1038/s41467-024-52282-w)
Supplement: Supplementary file 3 — Description of Additional Supplementary Files [file 41467_2024_52282_MOESM3_ESM.pdf]

Description of Supplementary Data file

Sheet “Plasmid list”

List and sequences of plasmids constructed in this study.

Sheet “Cloning primer list”

List and sequences of primers used in this study for plasmid cloning.

Sheet “Locus-specific primer list”

List and sequences of primers used in this study to validate locus-specific genomic modifications.
